# Evaluation of Shear Bond Strength of a Primer Incorporated Orthodontic Composite Resin: An In-Vitro Study

**DOI:** 10.7759/cureus.24088

**Published:** 2022-04-12

**Authors:** Rithika Joseph, Nausheer Ahmed, Abrar Younus A, K Ranjan R Bhat

**Affiliations:** 1 Department of Orthodontics and Dentofacial Orthopedics, Government Dental College and Research Institute Bangalore, Bangalore, IND

**Keywords:** ari score, transbond plus sep, gc ortho connect, bonding, shear bond strength

## Abstract

Introduction

Newer adhesive systems are available eliminating the separate priming step during the bonding procedure thereby reducing the chances of introduction of error during bonding. The purpose of this study was to compare the shear bond strength of a primer-incorporated adhesive with that of a self-etching primer system and conventional bonding system.

Materials and method

Sixty-six extracted human premolars were cleaned, mounted, and randomly divided into three groups. In group A (control), 22 teeth were bonded with stainless steel orthodontic brackets using the conventional bonding system; in group B, 22 teeth were bonded using a self-etching primer system (Transbond Plus SEP, 3M Unitek, Monrovia, CA) and in group C, 22 teeth were bonded using the new primer-incorporated adhesive system (GC Ortho Connect, GC Orthodontics, Breckerfeld, Germany). After bonding, the teeth were stored in artificial saliva at 37^º^C for 24 hours and debonded with a universal testing machine. The adhesive remnant index (ARI) was also evaluated. Statistical analysis was done using one-way ANOVA to compare the shear bond strength values among the three groups and Kruskal Wallis test was used for comparison of ARI scores.

Results

The SBS values in group A (11.60 ± 2.95 MPa), group B (9.44 ± 4.46 MPa) and group C (12.68 ± 6.25 MPa) were found to be comparable with no statistically significant difference. The ARI scores were also similar among the tested groups with the predominant site of bond failure being the bracket-adhesive interface indicating a safe bond-failure site.

Conclusion

GC Ortho Connect was found have clinically acceptable shear bond strength values that are comparable with that of self-etching primer and conventional bonding system. Therefore, it can be used effectively for saving the clinician’s chairside time by reduction in the number of steps during bonding without compromising on the bond strength.

## Introduction

In orthodontic practice, it is crucial for obtaining a reliable adhesive bond between an orthodontic attachment and tooth enamel. Innovations in bonding technology have led orthodontists to incorporate new adhesives, composite resins and bonding techniques into clinical practice [1]. The conventional bonding procedure involves three steps, enamel etching and priming, followed by the use of an orthodontic adhesive for bonding the bracket on teeth. From a clinical standpoint, longer bracket placement time may lead to difficulty in moisture control causing greater chances of bond failure and loss of valuable clinical time for the orthodontist [2]. As a result, bonding techniques that require fewer clinical steps have been introduced in the market.

Transbond Plus SEP (3M Unitek, Monrovia, CA) is a self-etching primer (SEP). When applied to the teeth, the etched surface is simultaneously primed eliminating the possibility of demineralized tissue that is incompletely infiltrated with resin [3].

GC Ortho Connect (GC Orthodontics, Breckerfeld, Germany) is a new light-cured orthodontic adhesive that incorporates the primer into the adhesive paste. As per the manufacturer guidelines, with GC Ortho Connect there is no need to apply primer on the tooth. Thus a simple etching of the enamel followed by drying is enough to prepare the enamel surface for the orthodontic bonding [4].

The nature of adhesive is significant in regard to the bond strength, residual adhesive on enamel and enamel damage. The shear bond strength of an orthodontic adhesive should be sufficient to withstand the masticatory forces on the orthodontic brackets, but it should not be too high which may cause enamel damage during debonding [5]. Therefore, an accurately scored adhesive remnant index (ARI) is an important consideration while selecting an orthodontic adhesive [6].

The aim of this in-vitro study is to determine if the new primer-incorporated composite can be used for improving the bonding procedure by minimizing working time during bonding and debonding without jeopardizing the ability to maintain clinically useful bond strength.

## Materials and methods

**Figure 1:**
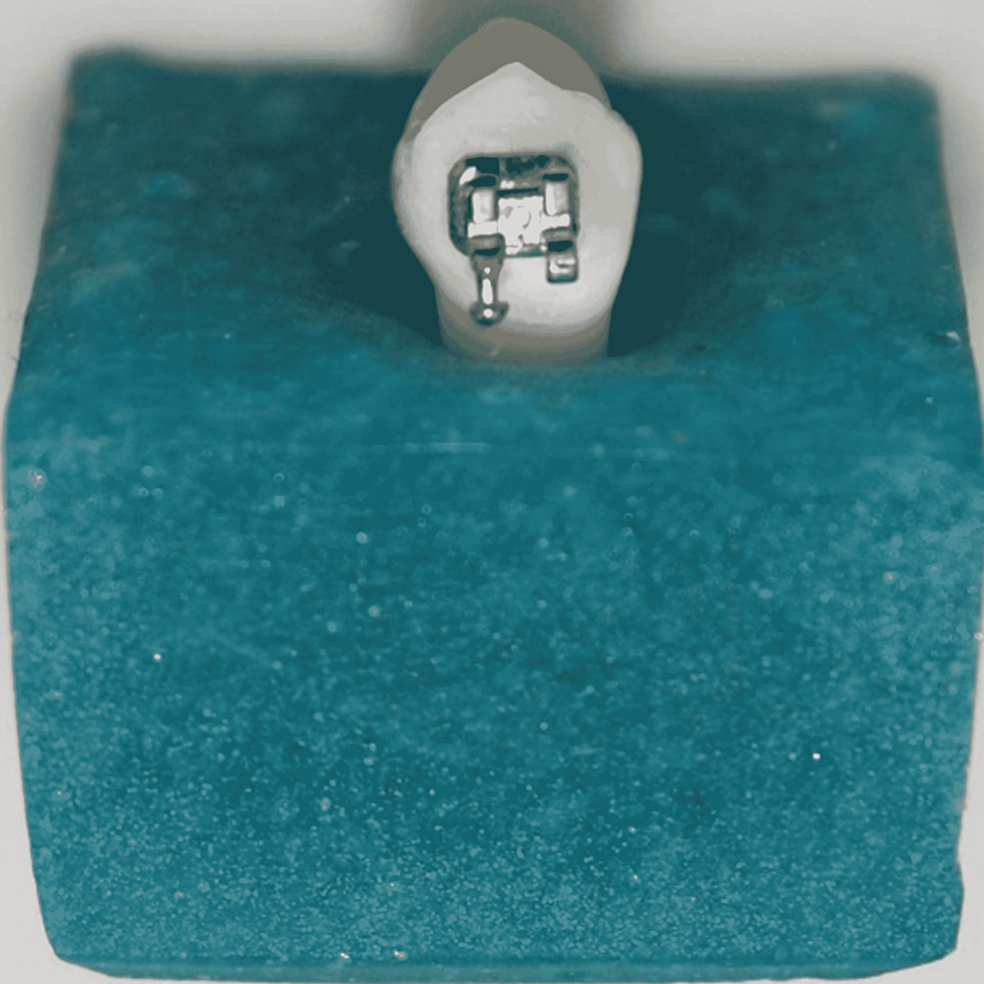
Tooth sample mounted on an acrylic block

Sixty-six human premolars extracted for orthodontic purposes were collected, cleaned, and stored in deionized water. Ethical clearance for collecting extracted human teeth was obtained from the Institutional Ethical Committee and Review Board, Government Dental College and Research Institute, Bengaluru (Approval number: GDCRI/IEC-ACM(2)/19/2019-20). Teeth with hypoplastic enamel, cracks, restoration, or caries were excluded. All teeth were separately mounted vertically in self-cure acrylic such that the crown was exposed as shown in Figure 1. The teeth were polished with a non-fluoridated flour of pumice using a rubber prophylactic cup for 10 seconds and then rinsed with a stream of water for 10 seconds.

**Figure 2:**
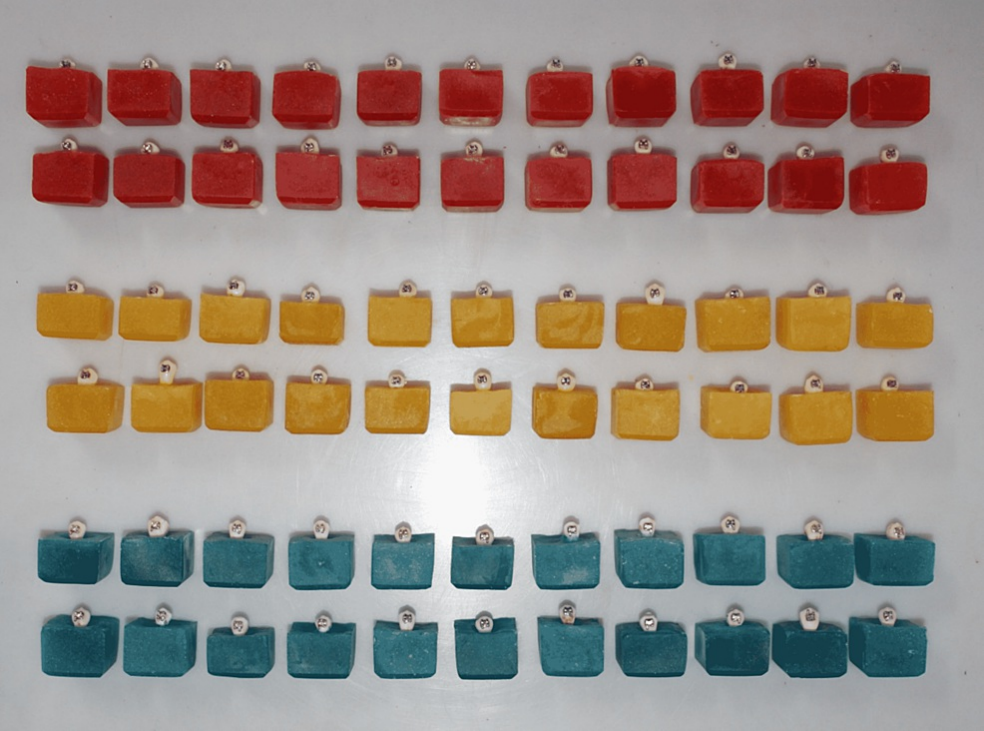
Sixty-six samples divided into three groups

The 66 premolar teeth were randomly divided into three groups with 22 samples in each group as shown in Figure 2. The brackets were bonded by three different protocols as mentioned below.

In group A (control), the teeth were etched with 37% phosphoric acid gel (ESPE, 3M Unitek, Monrovia, CA) for 30 seconds, rinsed and dried with oil-free compressed air for 20 seconds. Ormco Ortho Solo primer was then applied and light-cured on the etched enamel for 10 seconds followed by bonding of the bracket using Enlight LV (Ormco).

Group B was treated with Transbond Plus SEP (3M Unitek, Monrovia, CA) for 20 seconds, gently air-dried for 5 seconds and light-cured for 10 seconds. The brackets were then bonded using Enlight LV (Ormco).

**Figure 3:**
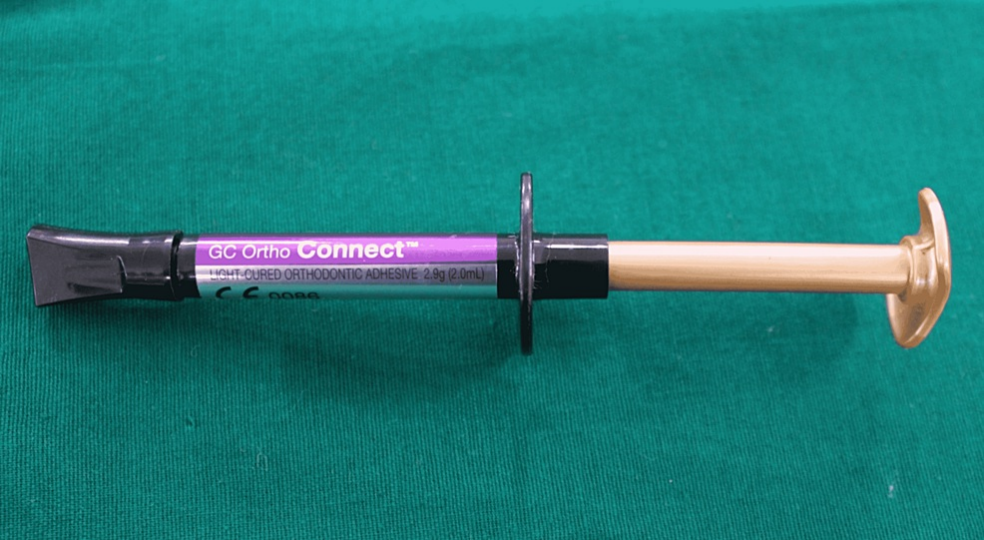
GC Ortho Connect

For group C, the teeth were etched with 37% phosphoric acid gel (ESPE, 3M Unitek) for 30 seconds, rinsed and dried with oil-free compressed air for 20 seconds. Brackets were then bonded using GC Ortho Connect (GC Orthodontics, Breckerfeld, Germany) (Figure 3).

0.022” slot MBT prescription stainless steel premolar brackets (Minimaster series, American Orthodontics) were used in all groups. All the brackets were cured for 30 seconds during bonding using Woodpecker i-LED light-curing unit.

**Figure 4:**
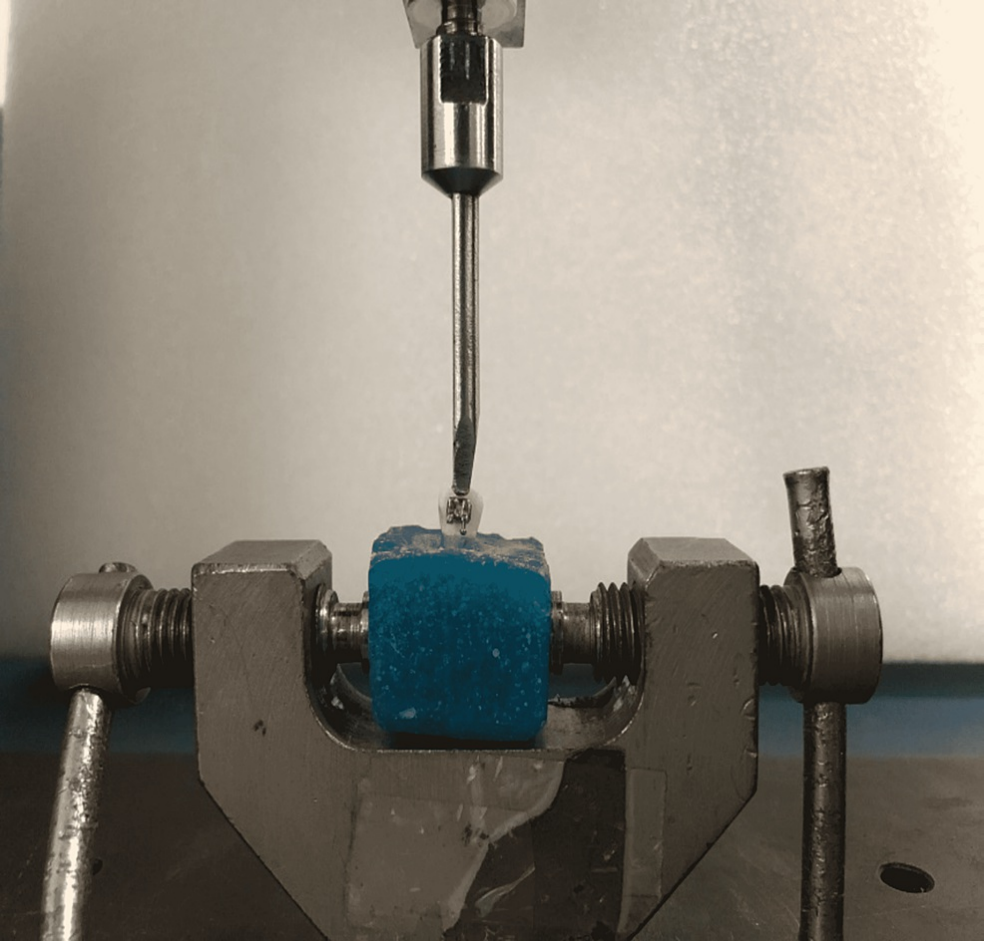
Sample mounted on UTM for shear bond strength testing

After bonding, all samples were stored in artificial saliva at 37ºC for 24 hours and then subjected to shear bond strength testing. Each tooth was oriented with the testing device as the guide such that its labial surface was parallel to the force during shear strength test as shown in Figure 4. A shear force was applied as close as possible to the tooth-bracket interface by a sharp chisel-shaped rod attached to the end of the testing machine at a predetermined crosshead speed of 5mm/minute until bracket failure [7]. A computer electronically connected to the Universal Testing Machine (MECMESIN, UTM Model 10) recorded the results of each test in Newton (N). The data were collected and converted into megapascals (MPa) with the following equation:

Shear force (MPa) = Debonding force (N)/(w/l);

Where, w= width of the bracket base, l = height of the bracket base, and 1 MPa= 1 N/mm^2^.

After debonding, the enamel surfaces were examined with a stereomicroscope at 10x magnification to score for ARI. The ARI scores range from 0 to 3: Score 0 - no adhesive left on tooth surface, Score 1 - less than half of the adhesive left on the tooth surface, Score 2 - half or more of the adhesive left on the tooth surface, Score 3 - entire adhesive left on the tooth surface [6].

Data were tabulated in Microsoft Excel 2010 and statistical analysis was performed. Descriptive statistics were given by mean, standard deviation, minimum and maximum values. Inferential statistics was done using one-way ANOVA for shear bond strength and Kruskal Wallis test for ARI. P-value less than 0.05 was considered statistically significant. Further, Pearson correlation was computed to relate shear bond strength to ARI score.

## Results

**Figure 5:**
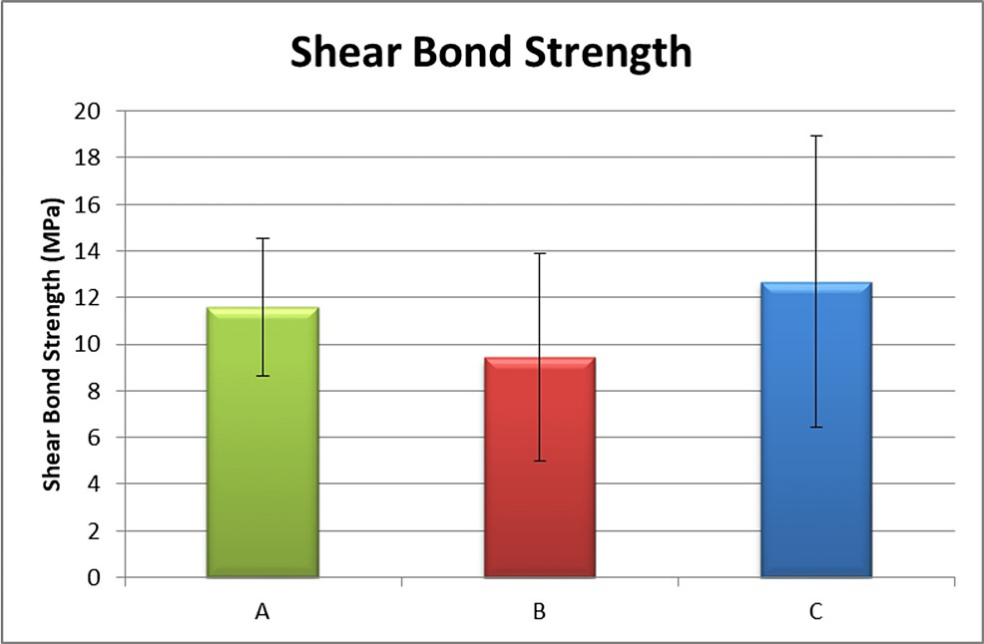
Shear bond strength comparison

The descriptive statistics for the shear bond strength of the tested groups are presented in Table 1. The highest mean shear bond strength was observed in the GC Ortho Connect group (12.68 ± 6.25 MPa) followed by the control group (11.60 ± 2.95 MPa) and the SEP group (9.44 ± 4.46 MPa). However, the difference between the three groups was not found to be statistically significant (p > 0.05). This indicated that the shear bond strength was comparable among the three groups (Figure 5). All the groups showed shear bond strength values that were clinically acceptable.

**Table 1:** Descriptive statistics for shear bond strength (MPa)

	n	Mean ± standard deviation	Range
Group A (Control)	22	11.60 ± 2.95	6.96 - 16.25
Group B (Self-etching Primer system)	22	9.44 ± 4.46	3.96 - 20.16
Group C (GC Ortho Connect)	22	12.68 ± 6.25	3.22 - 23.42

The frequency distribution of ARI scores for all the groups is shown in Table 2. The predominant failure of bond in all the groups was between the bracket and adhesive leaving half or more of the adhesive on the tooth surface, as shown by a score of 2. Score 0 was not shown by any of the tested samples, whereas scores 1 and 3 were noted in almost the same frequency among all the groups. The ARI scores indicate that all the tested bonding materials were essentially safe on the enamel during debonding.

Pearson correlation test was done to determine if there was any relation between the shear bond strength and the ARI. It was found that no such relation existed in our present study.

**Table 2:** Frequency distribution of ARI scores ARI - adhesive remnant index

	Score 0	Score 1	Score 2	Score 3
Group A	0	2	15	5
Group B	0	2	16	4
Group C	0	5	13	4

**Table 3:** Correlation between shear bond strength and ARI score ARI - adhesive remnant index

Estimate	Group A	Group B	Group C
Pearson's product-moment correlation sample estimate	-0.22	-0.08	-0.23
95 % confidence interval:	0.59 - 0.22	0.48 - 0.35	-0.59 - 0.21
t -statistic:	-1.03	-0.35	-1.07
p-value:	0.32	0.73	0.29
Interpretation	Not Significant	Not Significant	Not Significant

## Discussion

Direct bonding of orthodontic brackets has transformed and enhanced the practice of clinical orthodontics. Bonding of brackets was a major advancement from the traditional banding of teeth for orthodontic purposes that helped in significantly decreasing the occurrence of white spot lesions and reduced the chairside time during appliance placement. It also proved to be more esthetic and comfortable for the patient. However, further simplification of the bonding procedure is essential through evolution and improvement of existing methods without compromising the quality and effectiveness of the procedure.

In orthodontics, “shear” bond strength is assessed rather than peel, tension, torsion or cleavage because of its reproducibility and resultant reliability. Shear, tension, compression, and torsion (torque) are the most frequently used forces during debonding. As the force is applied at a distance from the bonding interface, shear tests usually involve a mix of shear and peel forces [8].

In this research, the shear bond strength of a primer incorporated adhesive system (GC Ortho Connect, GC Orthodontics, Breckerfeld, Germany) and a SEP adhesive system (Transbond Plus SEP, 3M Unitek, Monrovia, CA) has been compared with a conventional acid-etch bonding system (control).

The conventional bonding system that was followed in this study is a 3 step procedure. Acid conditioning of enamel with 37% phosphoric acid creates microporosities on the enamel surface, into which an unfilled resin (primer) is flown creating resin tags for mechanical retention. A filled resin (adhesive) is then used to attach the bracket to the prepared enamel surface. In the current study, the mean shear bond strength in the control group (conventional acid etch system) was found to be 11.6 MPa (Table 1).

Unlike conventional etchants, SEPs are not rinsed off from the tooth surface after application. Rather, all the minerals and smear layer are incorporated into resin that penetrates to the etched depth of enamel. The SEPs are designed to provide a continuous layer between the etched tooth surface and the adhesive by synchronously demineralizing and penetrating the tooth surface with acidic monomers that can polymerize in situ [3].

Many studies have shown that when SEPs are applied, the adhesive penetration into the etched enamel is less than when the traditional acid-etching approach is utilized. The further the acid penetration into the enamel surface, the larger the adhesive penetration and the greater is the risk of enamel damage during debonding [7]. This is an advantage of SEP over the conventional acid-etching system.

The SEP used in this study is Transbond Plus SEP (3M Unitek, Monrovia, CA). Results showed that the mean shear bond strength was 9.4 MPa (Table 1) for the samples bonded using Transbond Plus SEP under the test conditions. The SBS value of SEP was found to be less than that of the control group but it was not found to be statistically significant. This finding is in agreement with the study conducted by Grubisa et al. [9] who showed that the shear bond strength of the brackets bonded using Transbond Plus SEP was not statistically different from that obtained using 37% phosphoric acid and Enlight adhesive resin. Some studies have even reported significantly reduced shear bond strength of SEPs when compared with conventional acid-etch systems [10].

Tang et al. [11,12] proved through their in vitro and in vivo studies that bonding of brackets with or without primer application did not significantly affect the bond strength. However, there are some adhesive systems that incorporate primer into the adhesive paste.

In this regard, GC Ortho Connect (GC Orthodontics, Breckerfeld, Germany) is a light-cured adhesive that was initially launched in August 2013 in Japan under the name of Universal Bond. This light cured adhesive has primer incorporated in it, thereby eliminating the primer application step of the conventional bonding system. This may be critical while bonding the brackets as bracket placement requires more time and hence chances of moisture contamination is higher. The exclusion of a step also provides financial savings for the orthodontist and saves the chairside time. Moreover, eliminating the use of primer can also reduce the risk of leaving unpolymerized components, reducing cytotoxicity [8].

In this study, the mean shear bond strength of the brackets bonded using GC Ortho Connect was found to be 12.6 MPa (Table 1). This was higher than both the conventional and SEP groups but the difference was not found to be statistically significant. These results are in agreement with Shapinko et al. [4] who proposed that the shear bond strength of GC Ortho Connect was not statistically different from the bond strength of conventional adhesive system. They had also evaluated the efficacy of GC Ortho Connect when used with and without primer application for bonding orthodontic brackets and reported that it had no significant effect on the shear bond strength. Likewise, Bilen et al. [2] reported similar bond strength values when a conventional bonding system, SEP system and GC Ortho Connect were used for bonding brackets.

According to Reynolds et al., bond strength between 5.9 and 7.8 MPa has been found sufficient to withstand the typical orthodontic forces [13]. According to Murray et al. [14] , there is a potential difference between in vivo and in vitro bond strengths of an adhesive and the shear bond strength values were comparatively lower in vivo. This can support the findings of the current study in which all the groups tested had bond strength values that were above the optimum orthodontic bond strength Furthermore, the maximum bond strength of orthodontic attachments should be less than the enamel's breaking strength, which is around 14 MPa [1]. In this study, the bond strength shown by all the tested groups was found to be below this critical value showing that all the tested materials were essentially safe on the enamel.

Clean enamel surfaces have higher surface energy that allows for bonding, but fluoride on the surface can diminish the adherent's surface energy, limiting the adhesive's ability to spread [7]. In this study, all teeth were cleaned with non-fluoridated flour of pumice before bonding the bracket.

In the current study, it was observed that the shear bond strength of Transbond Plus and the GC Ortho Connect groups did not statistically differ (p > 0.05) from the control group. All the groups showed clinically acceptable bond strength values not exceeding the breaking strength of enamel. 

A lower ARI score indicates that the bond failure has occurred at the enamel-adhesive interface and very less adhesive is remaining on the enamel surface which reduces the time needed for removal of the adhesive from the tooth surface after debonding. But this has the potential danger of fracturing the enamel surface while debonding. Alternatively, a higher ARI score (bond failure at the bracket-adhesive interface or within the adhesive) leaves the enamel surface relatively intact but considerable chairside time may be required for removal of the residual adhesive after debonding [15].

The ARI scores as observed under 10x magnification using a stereomicroscope showed that the control, Transbond Plus group and the GC Ortho Connect group had a predominant score of 2 with no statistically significant difference (p > 0.05) (Table 2).

The correlation between the shear bond strength and ARI scores was also done in this study and was found to be not statistically significant (p > 0.05). This proves that there exists no relation between the shear bond strength and the ARI scores of an adhesive.

The thickness of the adhesive beneath each bracket plays a significant role in determining the shear bond strength. This film thickness was not standardized in this study which is a drawback. Another limitation was that the brackets were vaguely positioned in the center of the crown. The adaptation of the bracket on the tooth surface varies depending on its vertical and horizontal positioning, which in turn affects the shear bond strength of the bracket. 

## Conclusions

Within the limitations of the study, it was found that GC Ortho Connect had the highest shear bond strength and Transbond Plus SEP had the least shear bond strength among the groups tested. However, this difference was not found to have a statistically significant difference and was comparable with the control group. The ARI scores indicated that the bond failure had occurred mostly on the bracket-adhesive interface and the ARI scores among the tested groups were found to be comparable with no statistically significant difference. Furthermore, no correlation was found between the shear bond strength and the ARI.

Therefore, considering all the above-mentioned findings, it can be seen that the GC Ortho Connect and Transbond Plus SEP may be used clinically for the efficient bonding of orthodontic brackets. The usage of these materials instead of the conventional bonding system can help in saving the valuable clinical time of the orthodontist due to the reduction in the number of steps needed for the bonding procedure without compromising on the optimum orthodontic bond strength.
